# KMT2A::TBC1D5阳性早期前体T细胞急性淋巴细胞白血病1例

**DOI:** 10.3760/cma.j.cn121090-20250115-00031

**Published:** 2025-07

**Authors:** 昱瑛 李, 铎 徐, 瑞萍 胡, 杨 唐, 晓霞 赵, 晓亮 刘, 海 林, 素君 高

**Affiliations:** 吉林大学第一医院血液科，长春 130021 Department of Hematology, The First Hospital of Jilin University, Changchun 130021, China

患者，男，41岁，因“反复发热、咳嗽、咳痰1个月”于2024年6月19日来我院就诊。患者既往健康。血常规：WBC 5.5×10^9^/L、单核细胞比例20％、HGB 74g/L、PLT 140×10^9^/L。骨髓涂片：有核细胞增生活跃，原、幼淋巴细胞比例为75％；POX染色病理细胞100％阴性；外周血涂片：淋巴细胞比例增多至55％，原、幼淋巴细胞比例为12％。骨髓免疫分型：异常细胞占有核细胞比例为19.71％，主要表达CD7、胞质CD3（cCD3）、CD34、CD38、CD2、CD99、CD45RA^dim^；部分表达CD117、CD79a、CD33、CD25、CXCR4；不表达CD123、TCR、TCR4、CD19、CD20、CD10、CD5、CD4、CD8、CD1a、CD13、CD71、cMPO、CD57、CD56、CD3、CD45RO、HA-DR、nTDT，免疫表型符合早期前体T细胞急性淋巴细胞白血病（ETP-ALL）。染色体核型：46，XY[20]。多重PCR筛查64种融合基因阴性。基因突变筛查（二代测序）：Ⅰ类突变（具有强烈临床意义）：NOTCH1，c.4721T>C，p.L1574P，突变丰度8.38％；NRAS，c.38G>A，p.G13D，突变丰度4.83％。FISH：KMT2A基因重排占19.5％。全转录组mRNA测序检测融合基因：KMT2A::TBC1D5。KMT2A::TBC1D5新融合基因经实时逆转录PCR及Sanger测序得以验证。Sanger测序显示KMT2A基因的第8号外显子（NM_001197104.2）和TBC1D5基因的第19号外显子（NM_014744.2）之间存在融合。综上，患者被明确诊断为ETP-ALL伴KMT2A::TBC1D5融合基因阳性。

患者合并重症肺炎、体能状态差，难以耐受化疗，因此，于2024年6月24日予维奈克拉联合阿扎胞苷方案诱导治疗，治疗第21天复查骨髓涂片原、幼淋巴细胞比例为2％，流式细胞术检测T系微小残留病（MRD）阴性，疗效评价为骨髓无白血病状态，停用维奈克拉。患者于诱导治疗间歇第1天行腰穿，脑脊液脱落细胞学检测未见白血病细胞，流式细胞术检测异常T淋巴细胞比例为45.68％，考虑中枢神经系统白血病，予每周2次腰穿+鞘内注射治疗，共6次。治疗后脑脊液流式细胞术检测阴性。诱导治疗间歇第7天，复查骨髓涂片：有核细胞增生活跃，原、幼淋巴细胞比例为13.50％。流式细胞术检测T系MRD：表达CD7、CD34、cCD3的异常T淋巴细胞比例为3.62％。继续给予维奈克拉联合阿扎胞苷方案治疗。维奈克拉联合阿扎胞苷第2个疗程治疗第14天复查骨髓涂片：有核细胞增生活跃，原、幼淋巴细胞比例为4.00％。流式细胞术检测T系MRD：表达CD7、CD34、cCD3的异常T淋巴细胞比例为2.40％。维奈克拉联合阿扎胞苷第2个疗程治疗第28天复查骨髓涂片：有核细胞增生活跃，原、幼淋巴细胞比例为29.50％。流式细胞术检测T系MRD：表达CD7、CD34、cCD3异常T淋巴细胞比例为4.69％。考虑患者治疗效果不佳，且肺部感染好转、美国东部肿瘤协作组（ECOG）评分为1分，能耐受化疗，遂调整治疗方案为维奈克拉联合VDLD（长春新碱+柔红霉素+培门冬酶+地塞米松）方案，维奈克拉联合VDLD方案治疗第14天复查骨髓涂片：有核细胞增生活跃，幼淋巴细胞比例为1.00％。流式细胞术检测T系MRD：表达CD7、CD34、cCD3异常T淋巴细胞比例为0.39％。维奈克拉联合VDLD方案治疗第28天复查骨髓涂片为缓解骨髓象。流式细胞术检测T系MRD阴性。疗效评价为完全缓解。后予Hyper-CVAD-A（环磷酰胺+长春新碱+表阿霉素+地塞米松）方案巩固化疗1个周期，化疗间歇期复查骨髓涂片及T系MRD，疗效评价为完全缓解。患者于2024年11月15日行无关供者异基因造血干细胞移植（HLA：9/10），输注CD34^+^细胞8.4×10^6^/kg、单个核细胞7.34×10^8^/kg，+12 d粒系植入、+15 d血小板植入。截至2025年1月15日，患者已无病生存3个月余。

讨论：本例为成人ETP-ALL，初诊时染色体核型正常、融合基因筛查（包括常见的KMT2A基因重排）阴性，FISH检查发现KMT2A基因重排阳性，后行RNA-seq检查发现KMT2A::TBC1D5融合基因阳性，并经实时逆转录PCR及Sanger测序验证。本研究提示，传统细胞遗传学检测方法在鉴定罕见转录本时具有明显局限性，尤其在分子生物学检测阴性的情况下，FISH检测结合RNA-seq可提供更全面和准确的分子信息，用于明确KMT2A的重排形式及其相关伴侣基因。

